# Supplementary material for: TNFα Effects on Adipocytes Are Influenced by the Presence of Lysine Methyltransferases, G9a (EHMT2) and GLP (EHMT1)
Source: Biology (Basel). 2023 Apr 30;12(5):674. doi: 10.3390/biology12050674 (PMC10215715; doi:10.3390/biology12050674)
Supplement: Supplementary file 1 [file biology-12-00674-s001.zip › biology-2289197-supplementary.pdf]

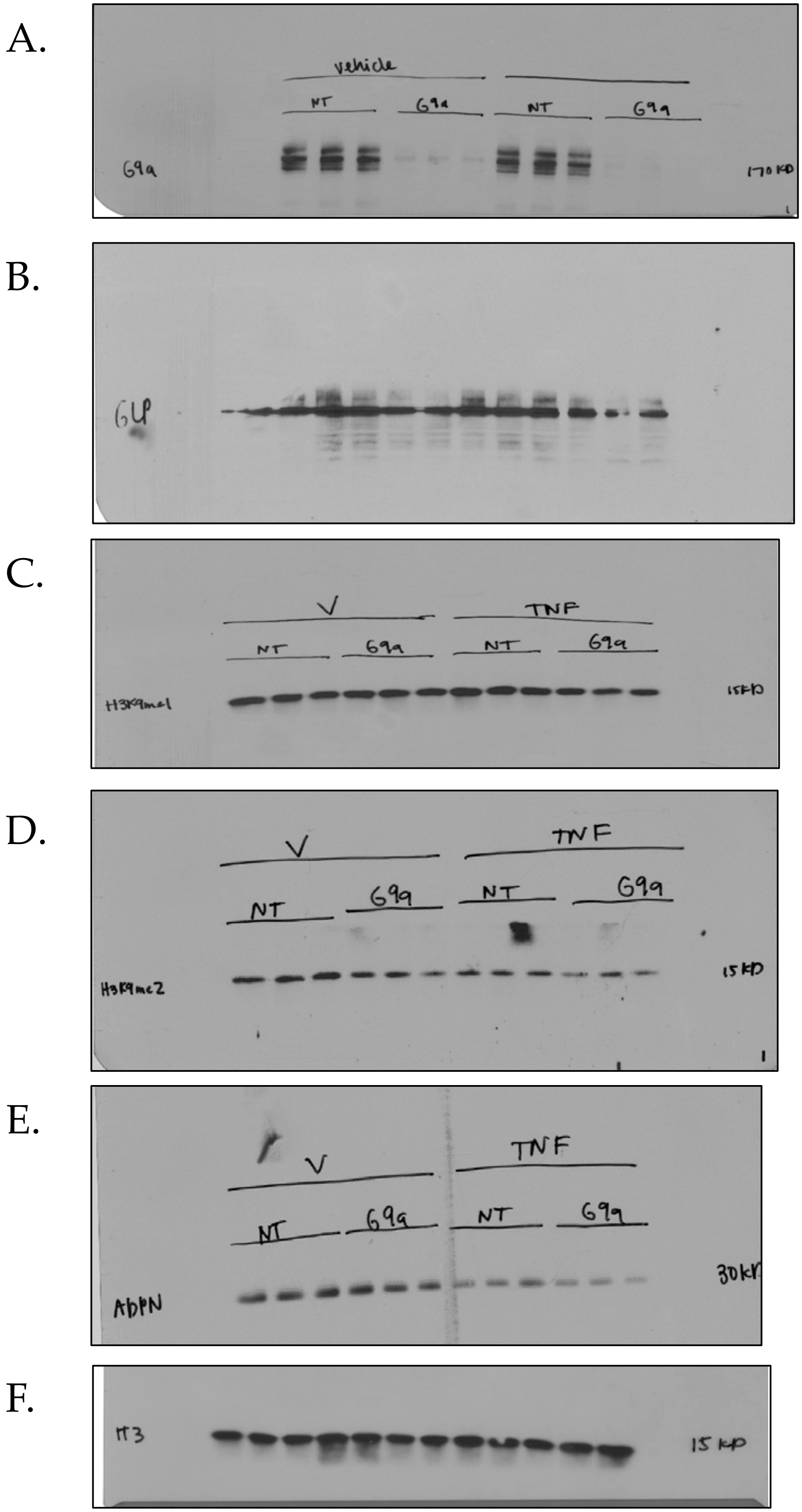

Figure S1. Original Western blot images related to Figure 1. **(A)** G9a, **(B)** GLP, **(C)** H3K9me1, **(D)** H3K9me2, **(E)** Adiponectin, and **(F)** Histone H3.

A.

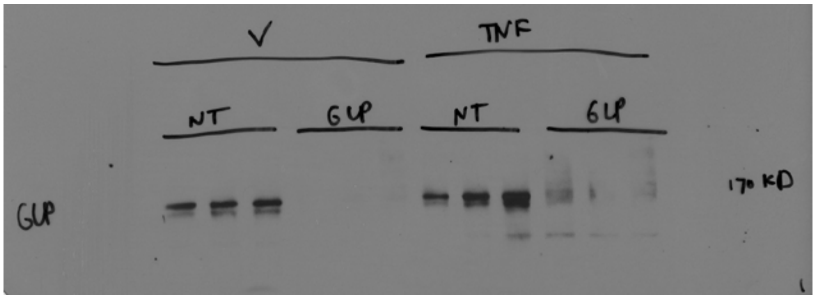

B.

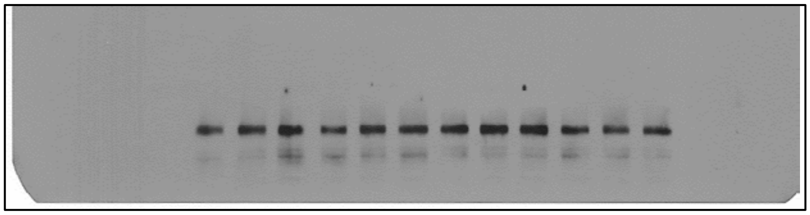

C.

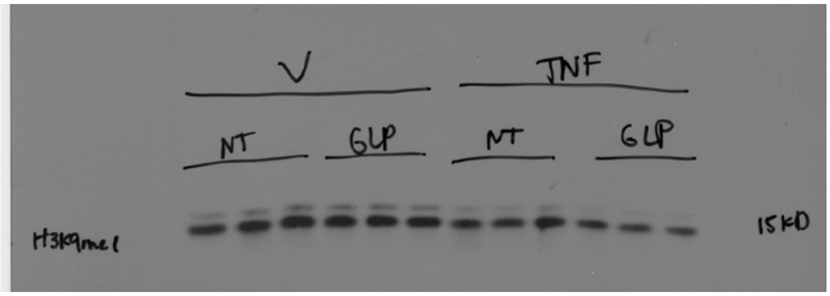

D.

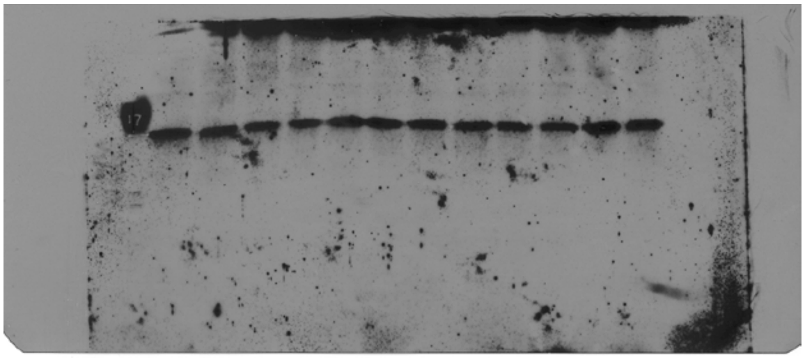

E.

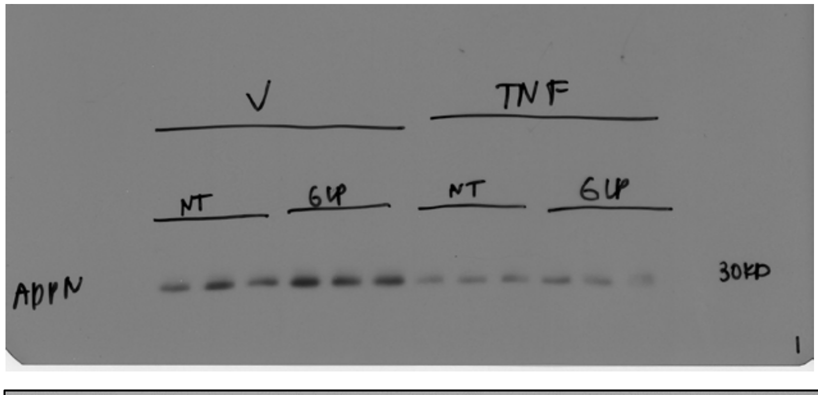

F.

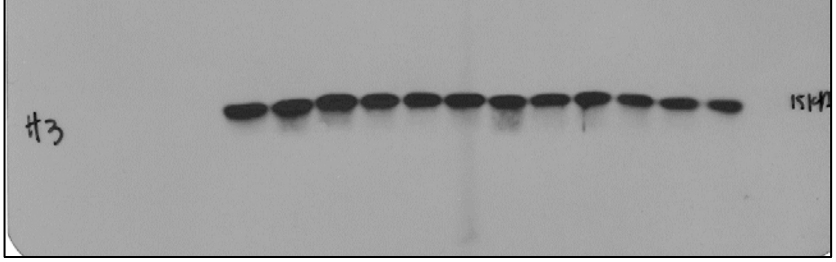

Figure S2. Original Western blot images related to Figure 2. **(A)** GLP, **(B)** G9a, **(C)** H3K9me1, **(D)** H3K9me2, **(E)** Adiponectin, and **(F)** Histone H3.

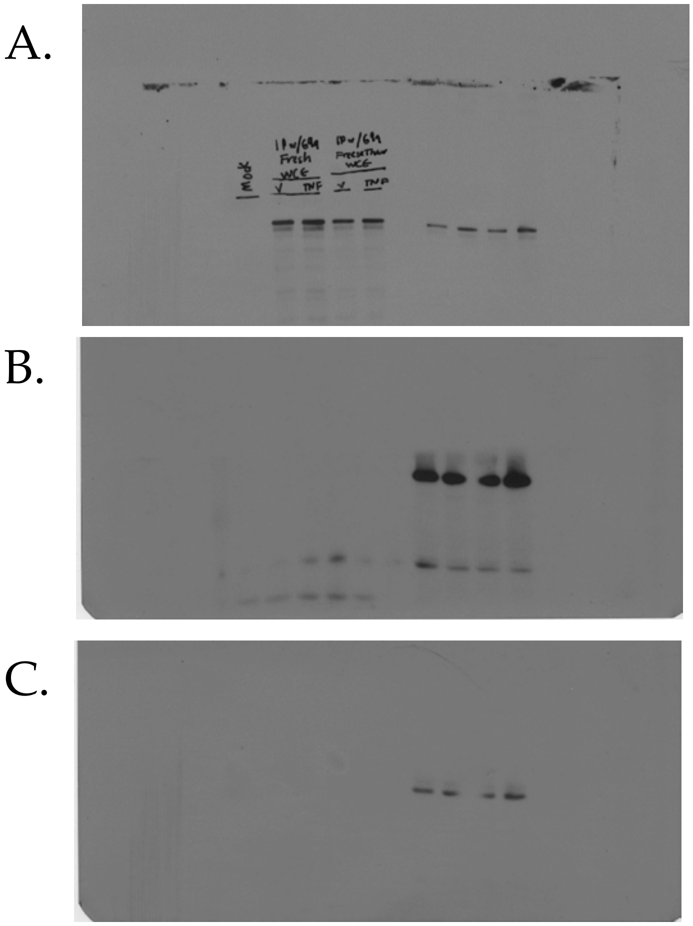

Figure S3. Original Western blot images related to Figure 4. **(A)** GLP, **(B)** STAT5A – dark exposure, and **(C)** STAT5A – light exposure.

A.

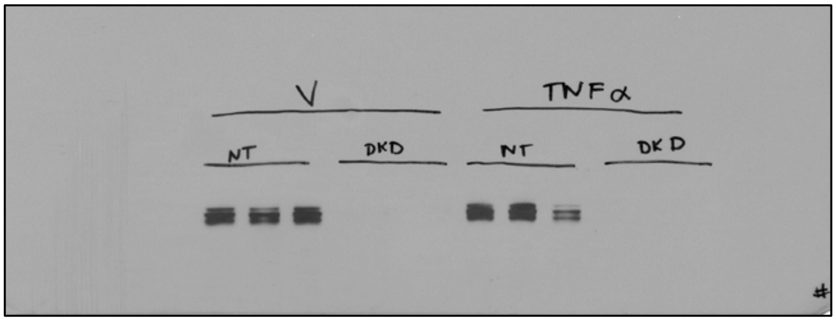

B.

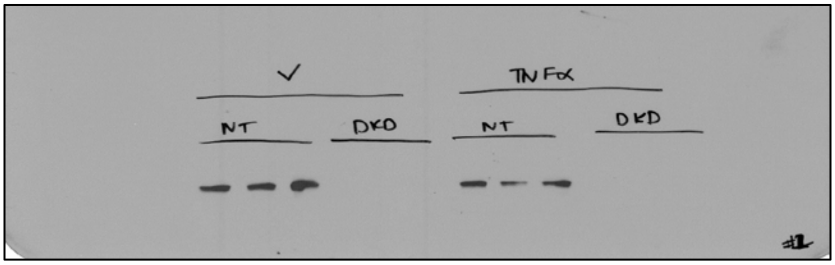

C.

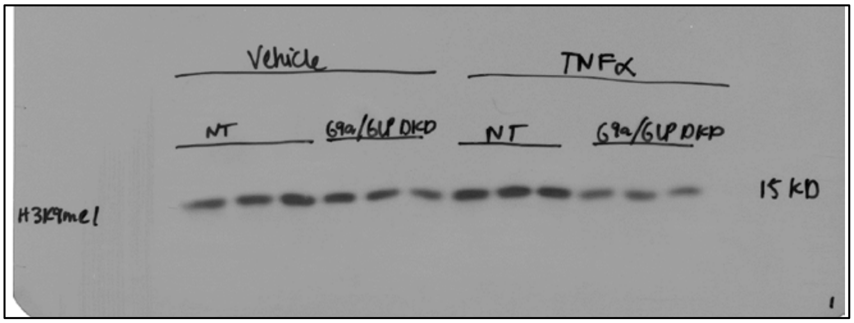

D.

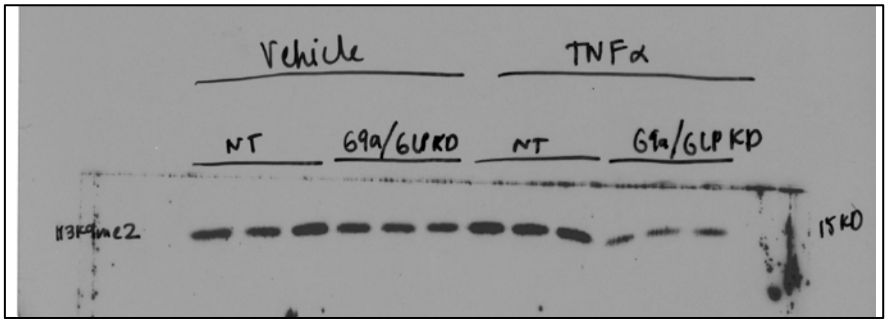

E.

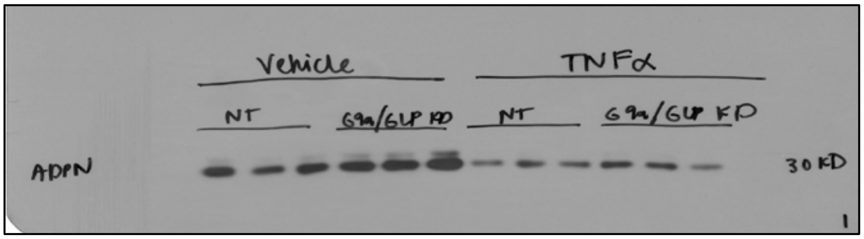

F.

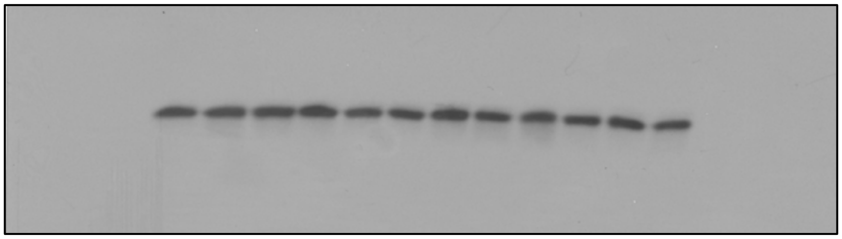

Figure S4. Original Western blot images related to Figure 5. **(A)** G9a, **(B)** GLP, **(C)** H3K9me1, **(D)** H3K9me2, **(E)** Adiponectin, and **(F)** Histone H3.

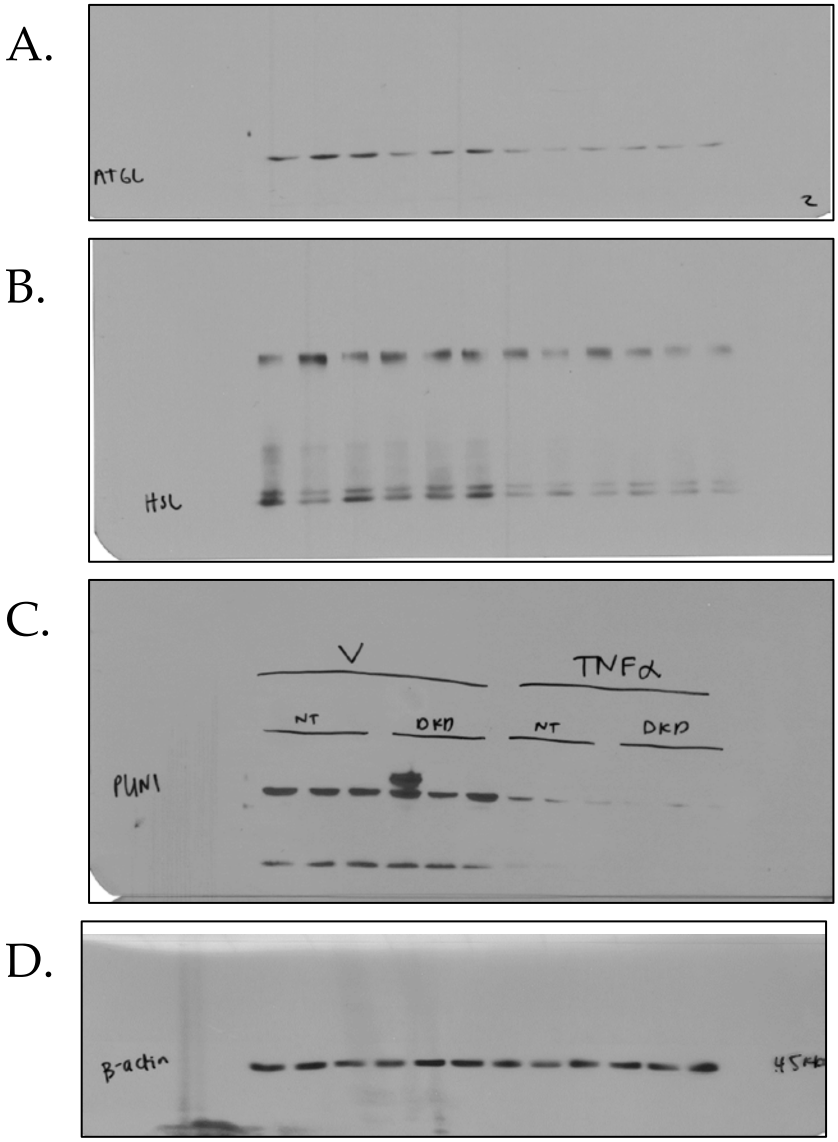

Figure S5. Original Western blot images related to Figure 8. **(A)** ATGL, **(B)** HSL **(C)** Perilipin, and **(D)** β-actin.

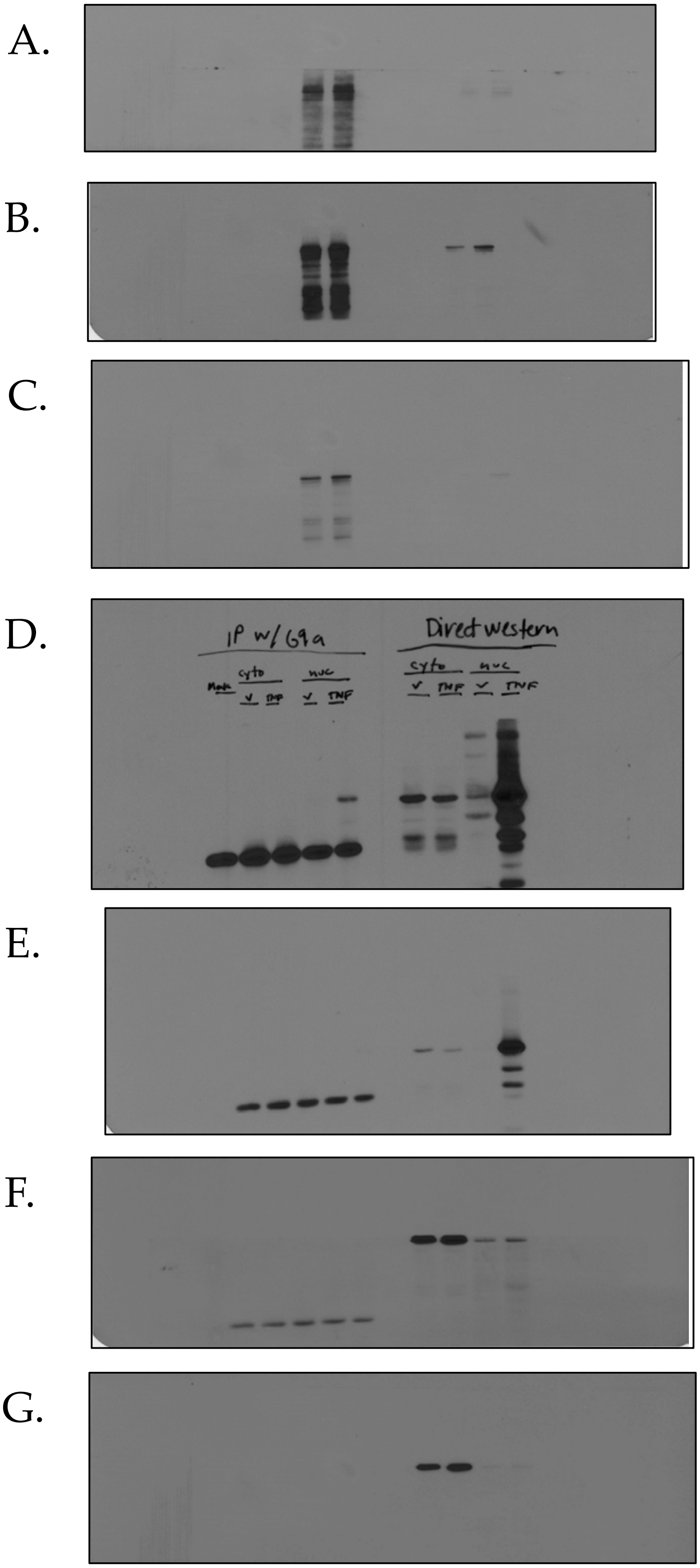

Figure S6. Original Western blot images related to Figure 9A. **(A)** G9a, **(B)** GLP – dark exposure, **(C)** GLP – light exposure, **(D)** p65 – dark exposure, **(E)** p65 – light exposure, **(F)** STAT5 – dark exposure, and **(G)** STAT5A – light exposure.

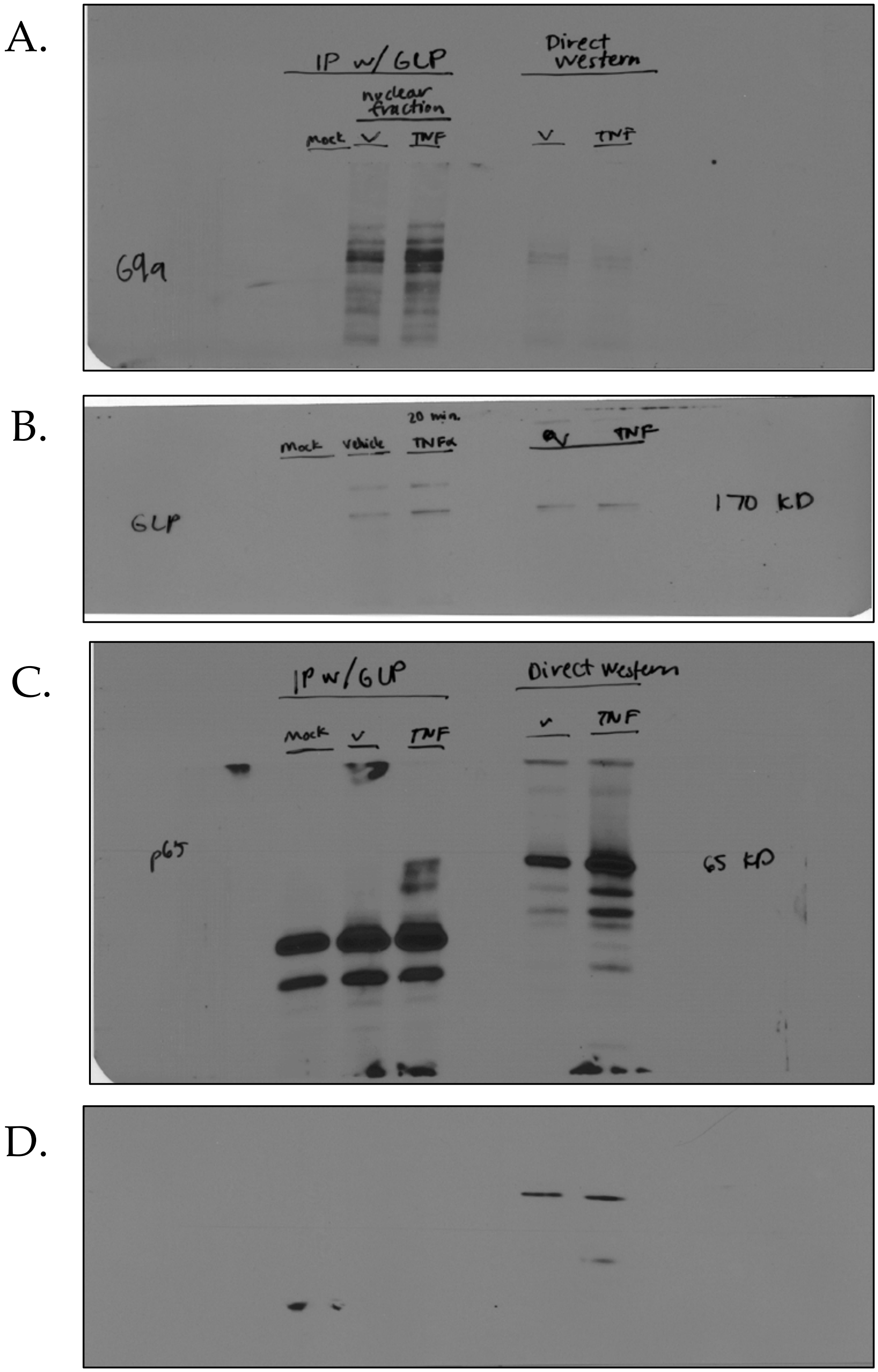

Figure S7. Original Western blot images related to Figure 9B. **(A)** G9a, **(B)** GLP, **(C)** p65, and **(D)** STAT5.
